# Supplementary material for: Grazing Effects of Soil Fauna on White-Rot Fungi: Biomass, Enzyme Production and Litter Decomposition Ability
Source: J Fungi (Basel). 2022 Mar 28;8(4):348. doi: 10.3390/jof8040348 (PMC9032049; doi:10.3390/jof8040348)
Supplement: Supplementary file 1 [file jof-08-00348-s001.zip › jof-1636581-supplementary.pdf]

Supplementary:

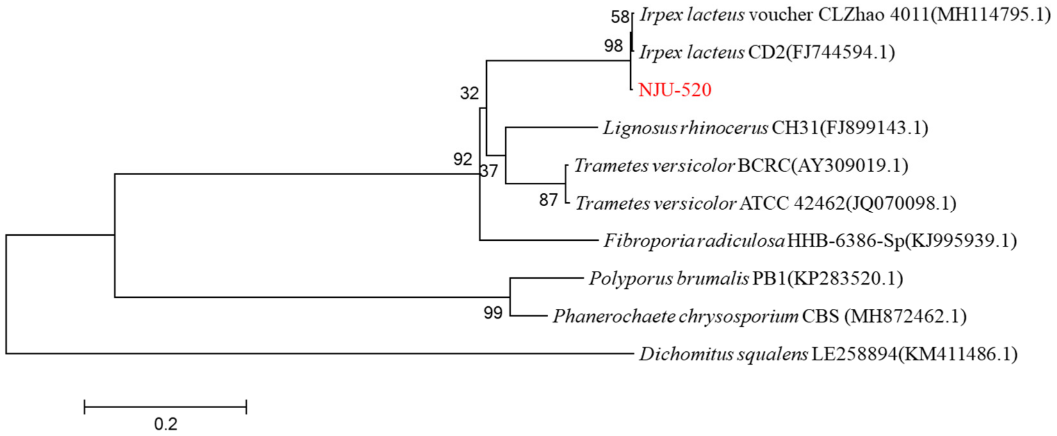

Figure S1. Construction of phylogenetic tree of NJU-520 and similar strains by neighbor-joining method. The GenBank accession numbers for each strain were listed in parenthesis. The phylogenetic tree was constructed by MEGA-X, and the scale bar represents 0.2 nucleotide substitution per nucleotide position.

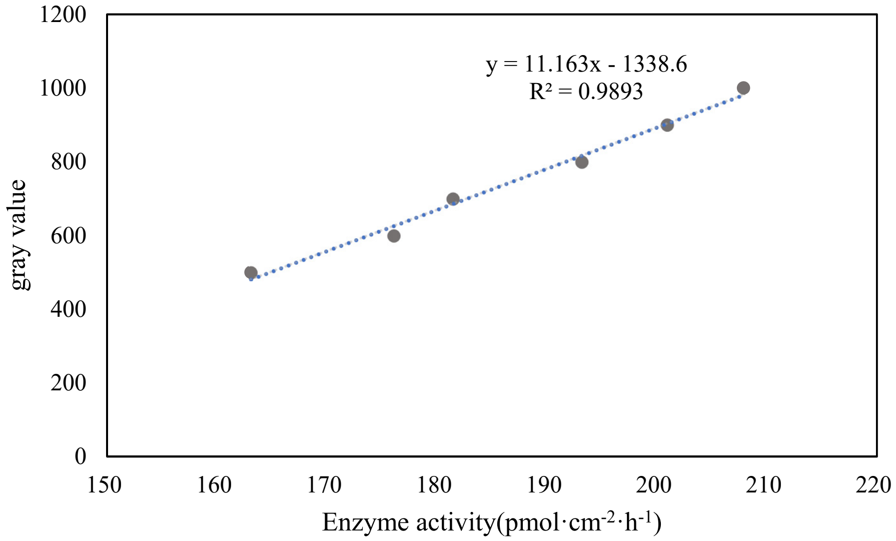

Figure S2. The standard curve of the correlation of zymography.

Table S1. The hotspots in the zymogram of the grazing area at 7 days, 21 days, 42 days and 70 days (%).

Enzyme	Treatment		Day 7	Day 21	Day 42	Day 70
Acid phosphatase	*Q. variabilis*	+Isopods	58.55±1.38ab	63.70±3.75bc	60.06±3.75bc	37.92±5.64a
		-Isopods	53.45±1.16a	50.12±3.89ab	43.13±1.56a	52.35±1.93a
	P. massoniana	+Isopods	62.50±1.43b	64.61±2.05c	49.37±2.45ab	48.92±1.60a
		-Isopods	57.13±0.40a	44.55±1.96a	45.52±0.83a	41.79±2.32a
β-N-acetylhexosaminidases	*Q. variabilis*	+Isopods	54.25±0.13a	66.40±3.42a	60.27±2.66b	61.40±2.05b
		-Isopods	54.56±0.67a	50.88±7.50a	46.72±2.65a	55.26±3.53b
	P. massoniana	+Isopods	53.09±1.69a	64.67±7.11a	60.80±2.12b	57.14±0.93b
		-Isopods	53.85±2.22a	41.17±5.48a	45.36±1.68a	42.58±1.80a

β-1,4-glucosidase	*Q. variabilis*	+Isopods	60.22±1.54a	66.18±4.11b	66.00±1.88b	62.48±3.03b
		-Isopods	56.29±2.01a	52.15±3.79ab	50.61±5.66a	55.03±4.24ab
	P. massoniana	+Isopods	59.10±3.04a	58.93±3.23ab	66.68±1.58b	61.32±0.81ab
		-Isopods	57.84±3.73a	45.50±5.80a	47.19±1.89a	48.14±2.58a

Table S2. List of chemicals

Chemical	Company	Producer
potato extract	Qingdao Hope Bio-Technology Co., Ltd.	hopebio
glucose	Sinopharm Chemical Reagent Co., Ltd.	SCR
agar	Guangzhou saiguo biotech Co., Ltd.	Biofroxx
aniline Blue	Shanghai yuanye Bio-Technology Co., Ltd.	Yuanye Bio-Technology
ammonium sulfate	Sinopharm Chemical Reagent Co., Ltd.	SCR
magnesium sulphate	Shanghai Macklin Biochemical Co., Ltd.	Macklin
potassium dihydrogen phosphate	Guangzhou Jinhuada Chemical Reagent Co., Ltd.	Guangdong Guanghua Sci-Tech Co., Ltd.
sodium carboxymethyl cellulose	Sinopharm Chemical Reagent Co., Ltd.	SCR
sodium chloride	Sinopharm Chemical Reagent Co., Ltd.	SCR
Congo Red	Beijing J&K Scientific Co., Ltd.	J&K Scientific
nalidixic acid	Shanghai yuanye Bio-Technology Co., Ltd.	Yuanye Bio-Technology
methanol	Sinopharm Chemical Reagent Co., Ltd.	SCR
petroleum	Shanghai Aladdin Biochemical Technology Co.,Ltd.	Aladdin
ethanol	Shanghai Macklin Biochemical Co., Ltd.	Macklin
p-nitrophenyl-β-D-glucoside	Nanjing Duly Biotech Co., Ltd	BioDuly
sodium carbonate	Xilong Scientific Co., Ltd.	Xilong Scientific
p-nitrophenyl-phosphate	Shanghai Kaiwei Chemical Co., Ltd.	Kaiwei Chemical
4-Methylumbelliferyl-β-D-glucoside	Shanghai Aladdin Biochemical Technology Co., Ltd.	Aladdin
4-Methylumbelliferyl-phosphate	Shanghai Aladdin Biochemical Technology Co., Ltd.	Aladdin
4-Methylumbelliferyl-N-acetyl-β-D-glucosaminide	ShangHai D&B Bioloqical Science and Technology Co., Ltd.	D&B
4-methylumbelliferone	Shanghai Aladdin Biochemical Technology Co., Ltd.	Aladdin
